# Fluorescence-Based Phenotypic Selection Allows Forward Genetic Screens in Haploid Human Cells

**DOI:** 10.1371/journal.pone.0039651

**Published:** 2012-06-22

**Authors:** Lidia M. Duncan, Richard T. Timms, Eszter Zavodszky, Florencia Cano, Gordon Dougan, Felix Randow, Paul J. Lehner

**Affiliations:** 1 Cambridge Institute for Medical Research, Addenbrooke’s Hospital, Cambridge, United Kingdom; 2 The Wellcome Trust Sanger Institute, The Wellcome Trust Genome Campus, Hinxton, United Kingdom; 3 MRC Laboratory of Molecular Biology, Division of Protein and Nucleic Acid Chemistry, Cambridge, United Kingdom; University of London, St George’s, United Kingdom

## Abstract

The isolation of haploid cell lines has recently allowed the power of forward genetic screens to be applied to mammalian cells. The interest in applying this powerful genetic approach to a mammalian system is only tempered by the limited utility of these screens, if confined to lethal phenotypes. Here we expand the scope of these approaches beyond live/dead screens and show that selection for a cell surface phenotype via fluorescence-activated cell sorting can identify the key molecules in an intracellular pathway, in this case MHC class I antigen presentation. Non-lethal haploid genetic screens are widely applicable to identify genes involved in essentially any cellular pathway.

## Introduction

Forward genetic analysis – the concept of identifying gene function from mutants with a discernible phenotype – has a proven track record of elucidating gene function. However, the difficulty in generating and recovering bi-allelic mutations in diploid cells is a major barrier to the application of this approach in the study of mammalian biology. In a major breakthrough, three groups recently circumvented this problem by pioneering forward genetic screens in haploid cells. Carette and colleagues [1] initially demonstrated the power of this approach by performing forward genetic screens in the near-haploid human KBM7 chronic myeloid leukemia cell line [2]. By creating a library of knockout KBM7 cells using a gene-trap retrovirus and screening for mutant cells resistant to a range of lethal insults, including bacterial toxins and cytotoxic viruses, they identified host genes required for toxin or viral killing [1], [3], [4]. More recently, two independent groups derived haploid murine embryonic stem cells lines and demonstrated the ability to perform forward genetic screens to identify genes required for sensitivity to toxins [5], [6].

In all these three examples, cells with mutations in relevant genes were isolated due to their resistance to a lethal insult. Such lethality screens are the simplest to perform but have inherent limitations. Few cellular processes are readily adaptable to a live/dead screen, and mutations that result in an intermediate phenotype are unlikely to be recovered. For haploid genetic screens to have widespread utility, a system is required whereby cells with relevant mutations can be selected based on a change in cell surface phenotype or the expression of a genetically-encoded reporter. The ability to screen on non-lethal phenotypes would greatly expand the scope of these methods such that they could be used to identify genes involved in essentially any cell-autonomous process.

Our aim was to test the applicability of this approach and determine whether cells with relevant mutations could be selected from a library of mutagenised near-haploid KBM7 cells on the basis of a change in cell surface phenotype. We chose to probe the cell surface expression of major histocompatibility complex class I (MHC-I) molecules, since the MHC-I antigen presentation pathway is well-characterized and utilizes a number of discrete components which function in a coordinated fashion to promote trafficking of peptide-loaded MHC-I molecules to the cell surface [7]. Newly-synthesized MHC-I heavy chains are co-translationally inserted into the endoplasmic reticulum (ER) where they heterodimerise with beta-2-microglobulin (β2m). MHC-I molecules then associate with the TAP peptide transporter (TAP1/2) via tapasin, a dedicated MHC-I chaperone which allows MHC-I access to TAP-delivered peptides generated from proteasome-mediated degradation of cytosolic proteins [7]. The successful loading of MHC-I molecules with peptide allows their release from the peptide loading complex and exit from the ER to the cell surface, where they present peptides to cytotoxic T lymphocytes (CTL). Here we show that KBM7 cells unable to present MHC-I molecules at the cell surface can be selected from a mutagenized library by fluorescence-activated cell sorting (FACS), and use this approach to isolate mutant clones deficient for components of the MHC-I antigen presentation pathway.

**Figure 1:**
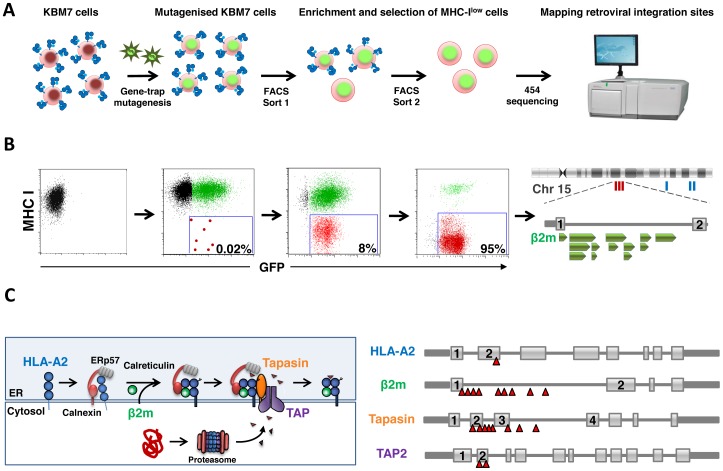
A haploid genetic screen to identify genes required for cell surface expression of MHC-I molecules. A. Schematic of the screen. **B.** Selecting MHC-I^low^ cells by FACS. Mutagenised KBM7 cells were labeled for surface MHC-I and those cells defective for MHC-I presentation enriched by two sequential rounds of FACS. The FACS plots correspond to the stages of the screen outlined above them in A. **C.** The genetic screen identifies multiple genes known to be involved in the MHC-I antigen presentation pathway. Important genes within the MHC-I antigen presentation pathway are targeted by multiple independent retroviral integrations (red triangles).

## Results

### A Haploid Genetic Screen to Identify Genes Required for Cell Surface MHC-I Expression

Near-haploid KBM7 cells express high cell surface MHC-I and HLA typing showed them to be HLA-A2, HLA-B60 and HLA-Cw10. These cells were mutagenised with a gene-trap retrovirus [1] to create a library of knockout cells, which were then selected by fluorescence-activated cell sorting (FACS) for the very small population of cells (∼0.02%) expressing low cell surface MHC-I (Fig. 1A). Two sequential sorts were required, with each sort enriching the selected population by at least two orders of magnitude. Following the first sort a small population of MHC-I^low^ cells was visualized (∼8%), which was further enriched to near purity during the second sort (Fig. 1B). Three separate screens were performed using different anti-MHC-I primary antibodies: w6/32, which recognizes total MHC-I, BB7.2, which is specific for HLA-A2, and 4E, which recognizes the HLA-B molecule. In each case, we selected for cells displaying reduced cell surface MHC-I. To identify the disrupted genes responsible for the defect in MHC-I expression, the retroviral integration sites in all sets of selected cells were amplified using splinkerette-PCR and sequenced using 454 pyrosequencing [8], [9]. Together this analysis revealed retroviral insertions in four genes known to be involved in the MHC-I antigen presentation pathway: 9 independent retroviral integrations were found in the gene encoding β2m, 8 in tapasin (TAPBP), 2 in TAP2 and 1 in the HLA-A2 gene itself (Fig. 1C). Overall, the identification of multiple known genes in the pathway demonstrates the power of this approach for interrogating the genetic basis of cellular processes.

### Generating Mutant Clones Deficient for Components of the MHC-I Antigen Presentation Pathway

**Figure 2:**
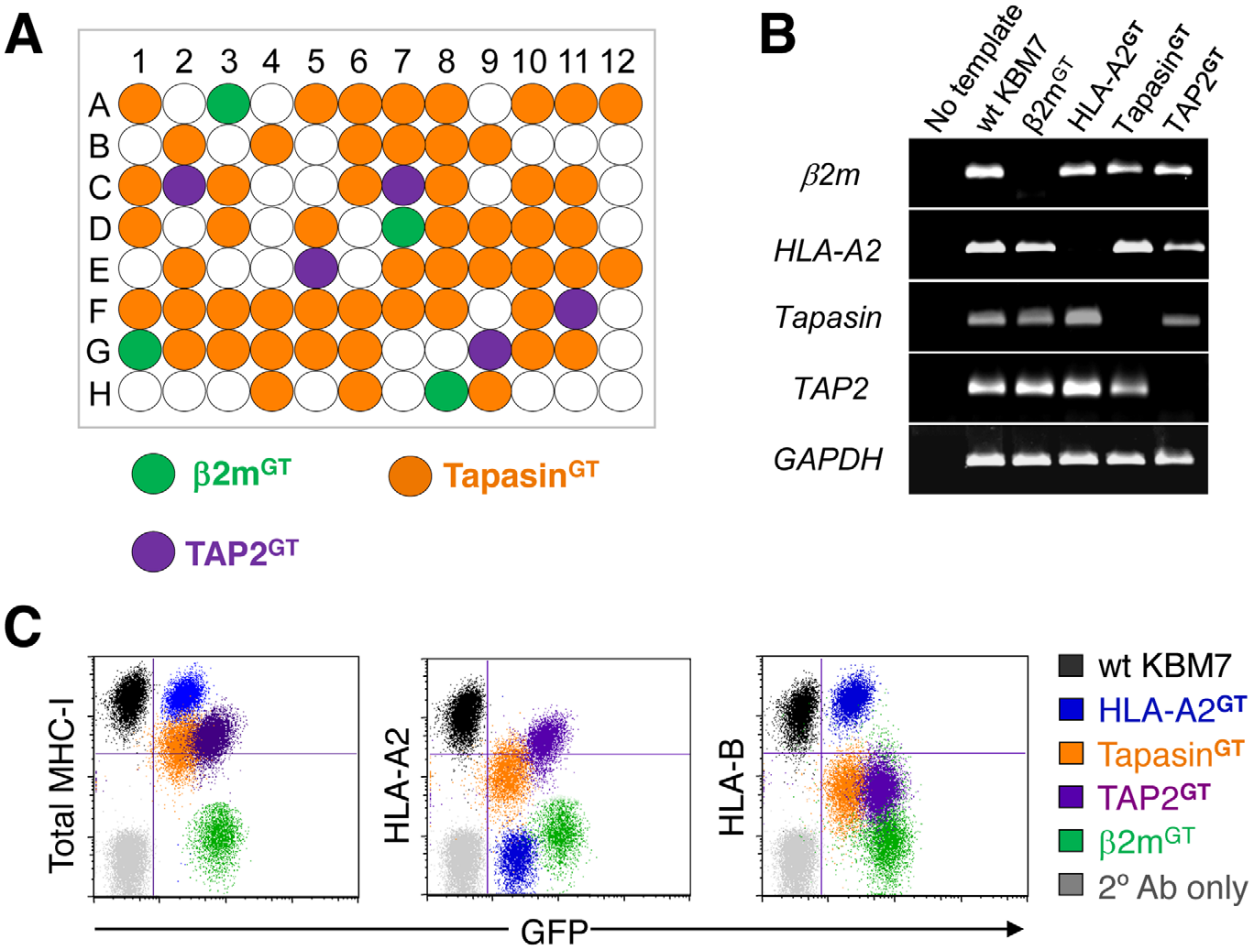
Isolation of knockout clones deficient for components of the MHC-I antigen presentation pathway. A. Identifying knockout clones by PCR. A schematic representation of the β2m, tapasin and TAP2 knockout clones identified by PCR from 96 single cell clones from the HLA-B^low^ selected population. Screening a relatively low number of single cell clones is sufficient to identify knockout cells representing the relevant target genes found in a screen. **B.** The gene-trap insertions result in a loss of gene expression. The knockout clones were analyzed for HLA-A2, β2m, tapasin and TAP2 expression by RT-PCR. **C.** Knockout of genes involved in the MHC-I pathway impairs cell surface expression of MHC-I molecules. The β2m, HLA-A2, tapasin and TAP2 knockout clones were labeled for the indicated proteins and analyzed by flow cytometry.

A major advantage of this technique is that genetically-deficient knockout human cells are generated as part of the screen, which therefore provides an invaluable resource to allow further functional characterization of the disrupted gene. To isolate knockout clones for the four genes involved in the MHC-I pathway, we plated single cells from the FACS-selected MHC-I^low^ populations into 96-well plates and identified the gene-trap insertion site in the resulting clones by PCR (Fig. 2A). Cells deficient in each of the four genes were recovered. The lack of expression of the disrupted genes was readily confirmed by RT-PCR (Fig. 2B), and we verified a functional knockout in each case by flow cytometry (Fig. 2C). Knockout of β2m abolished total cell surface MHC-I expression, while the knockout of HLA-A2 prevented only HLA-A2 surface expression, but left HLA-B60 and total MHC-I unaffected. Cells deficient in TAP2 and tapasin showed decreased surface MHC-I, with a more marked effect on HLA-B60 than HLA-A2, consistent with reports that the HLA-B heavy chain is more TAP-dependent since HLA-A2 can acquire TAP-independent, signal sequence-derived peptides [10], [11].

## Discussion

The enormous potential of forward genetic screens in cultured haploid mammalian cells is only tempered by their restricted use to live/dead screens. Here we show that selection on non-lethal phenotypes by FACS allows haploid genetic screens to be performed in human KBM7 cells leading to the identification of key components of an intracellular pathway. Fluorescent labeling and subsequent sorting for MHC-I^low^ cells among a library of mutagenised KBM7 cells led to the successful isolation of knockout KBM7 clones deficient in four components of the MHC-I antigen presentation pathway. This powerful approach can therefore be used to identify multiple components of an intracellular pathway. Furthermore, the iterative enrichment of cells expressing the desired phenotype by FACS, as opposed to screens based on live/dead phenotypes, has the additional advantage of identifying mutant cells with intermediate phenotypes (for example, as with the TAP2 and tapasin knockout cells) which are less likely to be identified in live/dead screens.

The efficient assembly of peptide loaded MHC-I molecules requires both MHC-I dedicated components (TAP, tapasin, the class I heavy chain and β2m) as well as more general cellular chaperones (calnexin, calreticulin and ERp57). While the majority of MHC-I dedicated components were identified in our screen, no mutations were found in genes encoding the more generalized cellular chaperones. Either the functions of these chaperones are redundant, or a growth disadvantage in cells deficient in these genes prevented clones from surviving the selection process.

Another solution to broaden the utility of haploid genetic screens is to convert an otherwise non-toxic screening agent into a lethal one to permit a live/dead screen. By attaching the catalytic subunit of diphtheria toxin to cholera toxin, which by itself is not lethal to KBM7 cells, Guimaraes and colleagues [12] identified new host genes required for cholera toxin intoxication. This approach is limited, however, as mutations will always be found in genes required for sensitivity to the toxic agent. Furthermore, mutations that confer only partial resistance are unlikely to be recovered. In contrast, the selection of mutant cells by FACS allows screens to be performed on a wide range of non-lethal phenotypes, and has the potential to identify mutations with an intermediate effect. This was the case in our MHC-I screens, as we successfully recovered cells deficient in TAP2 and tapasin, loss of which results in a partial rather than a total loss of cell surface MHC-I expression.

In summary, we report the success of FACS in allowing forward genetic screens to be performed in mutagenised haploid human KBM7 cells by enriching for cells of the desired phenotype. Although we have used this technique to select cells on the basis of a change in cell surface phenotype, this method is equally applicable to the selection of cells based on altered expression of a genetically-encoded fluorescent reporter. The ability to perform forward genetic screens in near-haploid KBM7 cells on non-lethal phenotypes opens up this technology for interrogating the genetic basis of a wide range of cellular processes.

## Materials and Methods

### Cell Culture

KBM7 cells [2] and HEK 293ET cells were maintained in Iscove’s Modified Dulbecco’s Medium (IMDM) supplemented with 10% fetal calf serum and 1% penicillin/streptomycin.

### Antibodies

The mAb W6/32 recognizes conformational MHC-I [13]; mAb BB7.2 (recognizes conformational HLA-A2) and mAb 4E (recognizes conformational HLA-B) were generous gifts from P. Cresswell. The Cy5-conjugated goat anti-mouse IgG secondary antibody was obtained from Jackson ImmunoResearch.

### Gene-trap Mutagenesis

The gene-trap vectors pGT0-GFP, pGT+1-GFP and pGT+2-GFP were a generous gift from T. Brummelkamp [1]. The bidirectional SV40 polyadenylation signal was replaced with a unidirectional one amplified as a BamHI-MluI fragment from the plasmid Flx800.15, to create pGT0-GFP-pA, pGT+1-GFP-pA and pGT+2-GFP-pA. Mutagenised KBM7 libraries were generated using a mixture of all three vectors.

Retrovirus was produced in HEK 293ET cells. HEK 293ET cells at greater than 90% confluency in a 6-well plate were transfected with 4 µg total DNA using Lipofectamine 2000 (Invitrogen). The gene-trap vectors plus the packaging plasmids pMD.GagPol and pMD.VSVG were mixed in a ratio of 10∶7∶3 in 250 µl OptiMEM (Invitrogen), mixed with 10 µl transfection reagent diluted in 250 µl OptiMEM, incubated for 20 min at room temperature and added dropwise to the cells growing in media without antibiotics. The virus-containing supernatant was harvested from 48 h post-transfection, filtered through a 0.45 µm filter, and applied directly to 1.5×10^6^ KBM7 cells in a 24-well plate in the presence of 10 µg/ml hexadimethrine bromide (Polybrene®, Sigma-Aldrich). The cells were then spun at 1800 rpm for 45 min, returned to the incubator, and after 3 h fresh media added.

### Flow Cytometry

Typically 2×10^5^ cells were washed with PBS, spun down (1600 rpm, 5 min, 4°C) and resuspended in 50 µl PBS. Primary antibody was then applied for 15 min at 4°C. The cells were then washed again with PBS, spun down, resuspended in 50 µl PBS and stained with a flurochrome-conjugated secondary antibody for 15 min at 4°C in the dark. Following a final wash step, the cells were fixed in 200 µl 0.3% formaldehyde and run on a FACSCalibur (BD Biosciences).

### FACS

∼5×10^7^ cells mutagenised cells were labeled with primary antibody for 15 min at 4°C in a total volume of 1 ml, washed with PBS, and then labeled with a Cy5-conjugated anti-mouse IgG secondary antibody in the same way. Sorting was carried out on an Influx cell sorter (BD Biosciences).

### Mapping of Retroviral Integration Sites and 454 Pyrosequencing

We employed a splinkerette PCR-based method to identify retroviral insertion sites, broadly as described by Koudijs and colleagues [9]. Briefly, genomic DNA was extracted from 1 million KBM7 cells (Gentra Puregene kit) and sheared by sonication (Bioruptor®, Diagenode) to an average fragment size of ∼400 bp. DNA fragment ends were blunted with T4 DNA polymerase (NEB), phosphorylated with T4 polynucleotide kinase (NEB) and ligated with annealed splinkerette adaptors (5′- GTTCCCATGGTACTACTCATATAATACGACTCACTATAGG-3′ and 5′- CCTATAGTGAGTCGTATTATAATTTTTTTTTCAAAAAAA-3′). Following digestion with BpmI (NEB), two rounds of PCR were carried out using one set of primers binding to the 5′LTR of the retroviral gene-trap vector and another set of primers binding to the splinkerette adaptor. For 454 pyrosequencing, the second round virus-end PCR primer included GS FLX 454 primer A plus a 10 bp barcode and the second round adaptor-end primer contained primer B. Primer sequences are detailed in Table S1. Multiplexing was achieved by amplifying samples from the different screens with unique barcode sequences; the DNA concentration of each sample following PCR amplification was quantified using a PicoGreen assay (Invitrogen) and mixed evenly. Following a column clean-up step (Qiagen PCR purification kit), the total library of PCR products was sequenced using the Lib-L emPCR kit (Roche) using one-quarter of a picotitre plate on a 454 GS FLX Titanium sequencing instrument (Roche). Processing of 454 reads was achieved using the Galaxy FASTX toolkit [14]. Reads were sorted by barcode, trimmed of viral and adaptor sequences, aligned to the human genome (hg19) using LastZ and visualized in IGV [15].

### Identification of Mutant Clones

FACS-selected MHC-I^low^ mutant cells were plated into individual wells of a 96-well plate. Three weeks later genomic DNA was extracted from the resulting clones and the site of the gene-trap insertion identified by PCR, using one primer (GFP_R) binding to the GFP in the gene-trap virus and the second primer binding at an appropriate distance from the insertion site within the HLA-A2, B2M, tapasin and TAP2 genes (Table S1).

### RT-PCR

Total RNA was extracted from KBM7 cells using the RNeasy Plus Mini Kit (Qiagen), and converted into cDNA by the addition of reverse transcriptase and an oligodT primer. A PCR reaction was then carried out using two primers binding within the mRNAs of the target genes designed to produce a ∼200 bp amplicon (Table S1). Products were visualized on a 2% agarose gel.

## Supporting Information

Table S1 **Primer sequences.**
(DOC)
