# Supplementary material for: Fluorescence-Based Phenotypic Selection Allows Forward Genetic Screens in Haploid Human Cells
Source: PLoS One. 2012 Jun 22;7(6):e39651. doi: 10.1371/journal.pone.0039651 (PMC3382162; doi:10.1371/journal.pone.0039651)
Supplement: Table S1 — Primer sequences. (DOC) [file pone.0039651.s001.doc]

**Supplementary Table 1**

| **Primer** | **Sequence 5’-3’** |  |
| --- | --- | --- |
| U3 LTR #6 | CTGATGGTTCTCTAGCTTGCCAAACCTAC | **First-round splinkerette-PCR primers** |
| Splink1 | gttcccatggtactactcata |
| U3 LTR #1-454A-MID | CCATCTCATCCCTGCGTGTCTCCGACTCAGACACATACGCCCAAACCTACAGGTGGGGTCTTTC | **Second-round splinkerette-PCR primers adapted for 454 pyrosequencing** |
| Splink2-454B | CCTATCCCCTGTGTGCCTTGGCAGTCTCAGTAATACGACTCACTATAGG |
| GFP_R | CAGCTCCTCGCCCTTGCTCAC | **PCR primers to identify retroviral insertions sites from genomic DNA extracted from single cell clones** |
| HLA-AB_Exon2 | GGTTCGACAGCGACGCCGCGAG |
| 5’β2m_Exon1  3’β2m_Exon2 | TCGCGCTACTCTCTCTTTCTG  AACTTCAATGTCGGATGGATG |
| 5’Tapasin_Intron1  5’Tapasin_Intron3  5’Tapasin_Exon3  3’Tapasin_Intron3 | GACATACAAACCGCTCCTCACTCG  GAAGTGCCCGGGTGGTAAGATTG  CCTGTTCTCATCACCATGGCAAC  CGAGACCACCGGCTGATCTGGACC |
| 5’TAP2_Exon2  3’TAP2_Exon2 | GTCGCTTCAGCCCCTTGGAGCTG  CAGCTCCAAGGGGCTGAAGCGAC |
| HLA-A2_RT-PCR_F | TCCTGCTACTCTCGGGGGCT | **RT-PCR primers to verify a loss of gene expression** |
| HLA-A2_RT-PCR_R | TCACTTTCCGTGCTCCCC |
| β2m_RT-PCR_F | TCGCGCTACTCTCTCTTTCTG |
| β2m_RT-PCR_R | AACTTCAATGTCGGATGGATG |
| Tapasin_RT-PCR_F | CGTCGCCACTCTAGCCCAAAG |
| Tapasin_RT-PCR_R | CTTGGCTGATGGTCAGCATATC |
| TAP2_RT-PCR_F | CACCTACACCATGTCTCGAATC |
| TAP2_RT-PCR_R  GAPDH_RT-PCR_F  GAPDH_RT-PCR_R | AGTTACTCATCAGGGTGGTATCC  TCCCATCACCATCTTCCAGG  ATGAGTCCTTCCACGATACC |

**Table S1. Primer sequences.** Primers sequences and PCR parameters for *HLA-A2* specific RT-PCR have been described [16].
